# Supplementary material for: Identification of 4-aminoquinoline core for the design of new cholinesterase inhibitors
Source: PeerJ. 2016 Jul 7;4:e2140. doi: 10.7717/peerj.2140 (PMC4941764; doi:10.7717/peerj.2140)
Supplement: Data S1 [file peerj-04-2140-s001.docx]

Raw data of figure 1: the raw data of inhibitory curve of compound **07** and **23** on AChE. The file can be opened by Graphpad 4.0.

Raw data of figure 2: the raw data of Lineweaver-Burk plots of compound **07**. The file can be opened by Graphpad 4.0.

Raw data of figure 3: the raw data of the binding mode prediction of **07** (A) and **23** (B) with AChE (PDB id: 2CKM). The file can be opened by Discovery studio 3.0.

Raw data of figure 4: the raw data of the binding mode prediction of **04** and **07** with AChE (PDB id: 2CKM). The file can be opened by Discovery studio 3.0.

Raw data of figure 5: the raw data of the structural determinants and modification strategy of 4-aminoquinoline core. The file can be opened by Chemdraw 11.0.
