# Supplementary material for: Identification of 4-aminoquinoline core for the design of new cholinesterase inhibitors
Source: PeerJ. 2016 Jul 7;4:e2140. doi: 10.7717/peerj.2140 (PMC4941764; doi:10.7717/peerj.2140)
Supplement: Supplemental Information 1 [file peerj-04-2140-s002.docx]

Identification of 4-aminoquinoline core for the design of new cholinesterase inhibitors

Yao Chen ^1, 2^, Yao-Yao Bian ^3^, Yuan Sun ^5^, Chen Kang ^6^, Sheng Yu ^1^, Ting-Ming Fu ^1, 2^, Wei Li ^1^, Yu-Qiong Pei ^1,^* and Hao-Peng Sun ^4,^*

^1^ School of Pharmacy, Nanjing University of Chinese Medicine, Nanjing, 210023, China;

^2^ Jiangsu Collaborative Innovation Center of Chinese Medicinal Resources Industrialization, Nanjing University of Chinese Medicine, Nanjing, 210023, China;

^3^ School of Nursing, Nanjing University of Chinese Medicine, Nanjing, 210023, China;

^4^ Department of Medicinal Chemistry, China Pharmaceutical University, Nanjing, 210009, China;

^5^ Department of Chemistry and Biochemistry, The Ohio State University, 43210, Columbus, Ohio, USA;

^6^ Division of Pharmacology, College of Pharmacy, The Ohio State University, Columbus, OH, 43210, USA;

***** Correspondence: [peiyuqiong@126.com](mailto:peiyuqiong@126.com) (Y.-Q. P.); [sunhaopeng@163.com](mailto:sunhaopeng@163.com) (H.-P. S.); Tel.: +86-25-8581-1524 (Y.-Q. P.); Tel.: +86-25-8330-1606 (H.-P. S.)

**Table S1.** The detailed information of the compounds in this study.

| **Cpd.** | **Serial Number** | **Purity, %** |
| --- | --- | --- |
| **01** | 718335 | 97 |
| **02** | 07336 | 99 |
| **03** | 411441 | 97.5 |
| **04** | A79205 | 97 |
| **05** | 275581 | 98 |
| **06** | 260789 | 98 |
| **07** | 05851 | 99 |
| **08** | 693944 | 95 |
| **09** | C70509 | 99 |
| **10** | 234318 | 98 |
| **11** | 174823 | 97 |
| **12** | N8141 | 98 |
| **13** | 178594 | 99 |
| **14** | 136107 | 99 |
| **15** | 909505 | 97 |
| **16** | N9005 | 99 |
| **17** | 166868 | 98 |
| **18** | A80009 | 99 |
| **19** | 525022 | 97 |
| **20** | A59654 | 97 |
| **21** | 630721 | 97 |
| **22** | 321752 | 97 |

**Table S2**. The Km and Vmax with errors from the non-linear regression fitting in Lineweaver-Burke plot of compound **07**.

| Concentration (nM) | 0 | 12.5 | 50 | 200 | 800 |
| --- | --- | --- | --- | --- | --- |
| Vmax (μM/min) | 0.28 ± 0.02 | 0.25 ± 0.02 | 0.17 ± 0.01 | 0.17 ± 0.01 | 0.17 ± 0.02 |
| Km (μM) | 71.37 ± 17.19 | 85.52 ± 22.49 | 120.30 ± 18.40 | 123.50 ± 29.90 | 195.80 ± 45.45 |
| R square | 0.96 | 0.95 | 0.98 | 0.96 | 0.97 |
