# Supplementary material for: Identification of 4-aminoquinoline core for the design of new cholinesterase inhibitors
Source: PeerJ. 2016 Jul 7;4:e2140. doi: 10.7717/peerj.2140 (PMC4941764; doi:10.7717/peerj.2140)
Supplement: Table S1 [file peerj-04-2140-s009.docx]

**Table S1.** The detailed information of the compounds in this study.

| **Cpd.** | **Serial Number** | **Purity, %** |
| --- | --- | --- |
| **01** | 718335 | 97 |
| **02** | 07336 | 99 |
| **03** | 411441 | 97.5 |
| **04** | A79205 | 97 |
| **05** | 275581 | 98 |
| **06** | 260789 | 98 |
| **07** | 05851 | 99 |
| **08** | 693944 | 95 |
| **09** | C70509 | 99 |
| **10** | 234318 | 98 |
| **11** | 174823 | 97 |
| **12** | N8141 | 98 |
| **13** | 178594 | 99 |
| **14** | 136107 | 99 |
| **15** | 909505 | 97 |
| **16** | N9005 | 99 |
| **17** | 166868 | 98 |
| **18** | A80009 | 99 |
| **19** | 525022 | 97 |
| **20** | A59654 | 97 |
| **21** | 630721 | 97 |
| **22** | 321752 | 97 |
