# Supplementary material for: Identification of 4-aminoquinoline core for the design of new cholinesterase inhibitors
Source: PeerJ. 2016 Jul 7;4:e2140. doi: 10.7717/peerj.2140 (PMC4941764; doi:10.7717/peerj.2140)
Supplement: Table S2 [file peerj-04-2140-s010.docx]

**Table S2**. The Km and Vmax with errors from the non-linear regression fitting in Lineweaver-Burke plot of compound **07**.

| Concentration (nM) | 0 | 12.5 | 50 | 200 | 800 |
| --- | --- | --- | --- | --- | --- |
| Vmax (μM/min) | 0.28 ± 0.02 | 0.25 ± 0.02 | 0.17 ± 0.01 | 0.17 ± 0.01 | 0.17 ± 0.02 |
| Km (μM) | 71.37 ± 17.19 | 85.52 ± 22.49 | 120.30 ± 18.40 | 123.50 ± 29.90 | 195.80 ± 45.45 |
| R square | 0.96 | 0.95 | 0.98 | 0.96 | 0.97 |
